# Impact of Bovine Lipocalin-2 Gene on the Antioxidant Activity of Milk from Polish Holstein-Friesian Cows

**DOI:** 10.3390/ani9110992

**Published:** 2019-11-18

**Authors:** Joanna Pokorska, Dominika Kułaj, Agata Piestrzyńska-Kajtoch, Anna Radko

**Affiliations:** 1 Department of Animal Reproduction, Anatomy and Genomics, University of Agriculture in Krakow, 30-059 Krakow, Poland; d.kulaj@ur.krakow.pl; 2 Department of Animal Molecular Biology, The National Research Institute of Animal Production, 32-083 Balice, Poland; agata.kajtoch@izoo.krakow.pl (A.P.-K.); anna.radko@izoo.krakow.pl (A.R.)

**Keywords:** antioxidant activity, milk, lipocalin-2, gene polymorphism

## Abstract

**Simple Summary:**

Recently, an increased interest in high health-value food products rich in natural antioxidants has been observed. Milk and milk products are one of the richest sources of biologically active compounds with antioxidant properties in products of animal origin. Lipocalin-2 (LCN2) is a small secreted protein, which is involved in inflammatory processes. Due to the fact that LCN-2 can be produced by various types of cells in response to oxidative stress and *LCN2* gene polymorphism has been associated with the somatic cells count in cow’s milk, the aim of this study was to validate the association of LCN2 polymorphism with antioxidant activity of milk from Holstein-Friesian cows. Four single nucleotide polymorphisms (SNPs) was identified, one of which g.98793763G>C was associated with higher antioxidant capacity in milk. The antioxidant capacity of milk also varied according to the age of cows, their daily milk yield, and somatic cell count (SCC) in milk.

**Abstract:**

In the recent years, antioxidant properties of food products have become an important aspect for consumers. Milk is a very good source of easily absorbable proteins and minerals, as well as a valuable source of antioxidants. Lipocalin-2 (LCN2), given that, inter alia, it is produced in large quantities by various types of cells in response to oxidative stress caused by physical or chemical factors, it can be considered a protein that determines the total antioxidant capacity of milk. The main objective of this study was to analyze polymorphisms within the lipocalin-2 gene and to determine their impact on antioxidant activity of milk from Holstein-Friesian cows. The genotyping was carried out by sequencing of PCR products. To determine the antioxidant activity of milk, the Trolox equivalent antioxidant capacity (TEAC) method was used. A total of four polymorphic sites were identified in the examined segment of the bovine lipocalin-2 gene. It was shown that cows of the CC genotype at the locus g.98793763G>C produced milk of significantly higher antioxidant capacity. The antioxidant capacity of milk also varied according to the age of cows, their daily milk yield, and SCC in milk.

## 1. Introduction

The contemporary lifestyle of people, which is dynamic, stressful, and often unhealthy, affects the body by giving rise to many unfavorable changes which lead to chronic diseases and promote aging processes. These changes are most often due to abnormal pro- and anti-oxidative homeostasis in the body. As a result, the recent years have seen consumers opting for foods rich in non-toxic, natural, and anti-oxidative substances as a key component of a diet oriented towards preventive health care.

Milk and dairy products are, first of all, the primary source of full-value proteins and minerals. As a product of animal origin, milk is also a very good source of antioxidants such as α-tocopherol, coenzyme Q10, conjugated linoleic acids (CLA), vitamins A and D, and phospholipids. The essential role of antioxidants in the body is to capture and neutralize free radicals which are produced in every cell during cellular respiration, but which are also released in greater quantities in response to attacks by pathogens, providing the fundamental mechanism of combating infections and repairing damaged tissues [1,2]. When an inflammatory reaction is triggered, free radicals (mostly reactive oxygen species (ROS) and reactive nitrogen species (RNS), but also free-radical metabolites of toxic substances) have destructive effects on cells of pathogens, thus causing their elimination. It is one of the fundamental mechanisms of innate immunity, which is observed e.g., in the mammary gland of cows suffering from clinical or subclinical mastitis [3]. Inflammatory reaction increases the level of granulocytes (in particular neutrophils and macrophages), which are responsible for generating greater quantities of free radicals necessary to neutralize pathogens in the process of phagocytosis, as well as for producing proteins which protect epithelial cells from potentially harmful products of heavy oxidative stress. One of these proteins is lipocalin-2 (LCN2, NGAL—neutrophil gelatinase-associated lipocalin). This protein belongs to the superfamily of lipocalins, small secretory proteins which are responsible for transporting small hydrophobic substances [4]. LCN2 may be present in the form of a 25-kDa monomer, a 46-kDa homodimer, or it can be joined by a covalent bond with metalloproteinase (MMP-9) in granules of neutrophils [5]. In the course of mastitis, LCN2 is released from granules of neutrophils, binds to iron molecules, and transports them to cells of the innate immunity system [6]. In some bacteria, the capability of sourcing iron is one of the central elements underlying their pathogenic properties. For many pathological bacteria, the limited availability of iron is a signal that stimulates them to synthesize siderophors which help them access this element [7]. The fact that LCN2 binds to iron, thus reducing the level of free iron ions which otherwise might be used in the process of bacterial growth, makes LCN2 a natural bacteriostatic [8]. It has also been demonstrated that LCN2 acts as “a stress protein”, being produced in very large quantities by various types of cells in response to oxidative stress caused by physical and chemical factors [9]. The bovine lipocalin-2 gene (Gene ID: 526639, NCBI, Bethesda, MD, USA) consists of 7 exons and 6 introns, within which multiple polymorphisms were identified, including, in particular, single nucleotide polymorphisms (SNPs). Some of these polymorphisms have been associated with production traits of Holstein-Friesian cattle [10]. To date, there have been no reports in the literature regarding the association of the *LCN2* gene polymorphisms and the antioxidant activity of cows’ milk, and therefore the principal objective of this study was to analyze polymorphisms within a segment of the promoter sequence, exon 1 and a fragment of intron 1 of the lipocalin-2 gene, and to determine their impact on the antioxidant activity of milk from cows of the Holstein-Friesian breed, black-and-white variety.

## 2. Materials and Methods

### 2.1. Samples Collecting

The test material comprised milk samples collected from 169 black-and-white Holstein-Friesian cows from one of the factory cattle farms in south-western Poland. The animals were kept in a free stall system and fed a total mixed ration (TMR). The cows originated from 84 bulls. Milk samples were collected into containers of 100 mL capacity in accordance with the ethical principles approved by the National Ethical Committee for Experiments on Animals at the Ministry of Science and Higher Education in Poland (2938/2015) and stored at a temperature of 4 °C until tested. The data on milk performance (daily milk yield, fat, protein and lactose levels, and somatic cell count (SCC)) of the analyzed cows were acquired based on daily milk yield reports recorded in a computerized herd management system.

### 2.2. SNPs Genotyping

The genotyping was carried out using DNA obtained from somatic cells of milk and the DNA isolation method developed by Pokorska et al. [11]. Subsequently, the PCR reaction was performed to replicate a 592-bp segment of the bovine lipocalin-2 gene (composed of a portion of the promoter sequence, exon 1, and a fragment of intron 1) using the primers suggested by Pokorska et al. [10]. Once PCR products were purified enzymatically using EPPiC (A&A Biotechnology, Gdynia, Poland), they were processed by sequencing using a BigDye™ Terminator v3.1 Cycle Sequencing Kit (Applied Biosystems) in accordance with the procedure described by Pokorska et al. [10]. All the results and SNP positions were analyzed using Variant Reporter Software 2 (Applied Biosystems) and BioEdit Sequence Alignment Editor [12], in reference to NC_037338.1, Bos taurus breed Hereford chromosome 11, ARS-UCD 1.2, whole genome shotgun sequence.

### 2.3. Determination of the Antioxidant Activity of Milk

The antioxidant activity of milk was determined using the TEAC (Trolox equivalent antioxidant capacity) method, which is utilized for the determination of the total antioxidant capacity of biological and food samples based on the level of absorbance [13]. For this purpose, extracts from milk samples were used, homogenized with methanol at a ratio of 1:10. To generate free radicals as necessary to use the TEAC method, the primary reagent was prepared 16 h before performing the TEAC test in that ABTS (2,2’-azino-bis(3-ethylbenzothiazoline-6-sulphonic acid)) was dissolved in distilled water to achieve a concentration of 7 mM, and then persulphate potassium was added to achieve the final concentration of 2.45 mM. After 16 h of incubation of the primary reagent in darkness, the test reagent was prepared by diluting the primary reagent with 96% ethanol. Absorbance was measured at a wavelength of 734 nm using a UV-VIS Genesys 20 (Thermo Scientific TM, Thermo Fisher Scientific, Waltham, MA, USA) spectrophotometer. To eliminate the effects of the solvent on the final test results, 30 μL of methanol was mixed with 3 mL of the test reagent and absorbance was measured after 30 min. Subsequently, the anti-oxidative activity was determined for milk extracts (30 μL of extract was mixed with 3 mL of the test solution) by measuring absorbance after 30 min for two replicates. The results are presented in terms of antioxidant activity given in μM TE (Trolox equivalent)/mL [13,14].

### 2.4. Statistical Analysis

The frequencies of alleles and genotypes were estimated for the identified SNPs in accordance with the guidelines developed by Falconer et al. [15]. The level of genetic equilibrium for all the analyzed loci was assessed using Hardy–Weinberg equilibrium calculator [16]. Additionally, the effective number of alleles (Ne), gene heterozygosity (He), and polymorphic information content (PIC) were estimated in accordance with the guidelines described by Nei and Roychoudhury [17]. The linkage disequilibrium (LD) level between the analyzed SNPs, given by the parameters D’ (so-called standardized disequilibrium coefficient) and r2, was determined using Haploview 4.2 software package [18].

The impact of genotypes within the identified loci of SNPs in the lipocalin-2 gene and the age of cows on milk traits such as the antioxidant capacity of milk, daily milk yield, percentage of fat, protein, lactose, and SCC in milk was assessed using the PROC GLM procedure of SAS^®^ 9.4 software in combination with Scheffe’s test (SAS Institute, Cary, NC, USA) according to the following animal model:

Y_ijk_ = µ + G_i_ + A_j_ + ε_ijk_, (1)

where Y_ijk_—milk trait; µ—general mean; G_i_—fixed effect of ith single SNP genotype of *LCN2*; A_j_—age effect (j = ≤4 years, 4.1–6.0 years, >6.0 years); ε_ijk_—random error.

Additionally, based on a multi-factor analysis of variance, the impact of the daily milk yield, percentage of protein, fat, lactose, and SCC in milk on the antioxidant capacity of milk was determined. The following statistical model was used for this purpose:

Y_ijklmn_ = µ + B_i_ + C_j_ +D_k_+ F_l_ + H_m_ + ε_ijklmn_, (2)

where Y_ijklmn_—antioxidant capacity of milk; µ—overall mean; B_i_—effect of ith daily milk yield (kg) (≤25.0, 25.1–35.0, >35.0); C_j_—effect of jth milk fat percentage (≤3.20, 3.21–4.00, >4.00); D_k_—effect of kth protein percentage (≤3.50, 3.51–4.00, >4.00); F_l_—effect of lth lactose percentage (≤4.40, 4.41–4.80, >4.80); H_m_—effect of mth SCC (×10^3^ cells/mL) (<200, 200–400, >400); ε_ijklmn_—random error.

## 3. Results

A total of four polymorphic sites were identified in the examined segment of the bovine lipocalin-2 gene, which have already been provided with SNP reference numbers (g.98793626A>G—rs 436227463, g. 98793763G>C—rs 133552604, g.98793812C>T—rs 135269094, and g.98793889G>A—rs 132900291). The first SNP is located in the promoter sequence and the other three in exon 1 of the *LCN2* gene. In the positions 98793763G>C and g.98793812C>T, there are silent mutations, while the mutation g.98793889G>A is a missense mutation which transforms arginine (AGG) into lysine (AAG) in position 36 of the amino acid chain of lipocalin-2. The analysis of the linkage disequilibrium demonstrated a 100% linkage between the loci g.98793626A>G and g.98793812C>T (D’ = 1, r^2^ = 1), which had been described by Pokorska et al. (2019). In case of other analyzed loci, it was did show linkage between them. Therefore, only three loci of SNPs (g.98793626A>G, g.98793763G>C, and g.98793889G>A) were taken into account in further statistical analysis. The frequencies of the identified genotypes and alleles, as well as the genetic diversity parameters of the genotyped SNPs within the *LCN2* gene, are shown in Table 1. It was determined that the herd of Holstein-Friesian cattle analyzed as part of this study presented genetic disequilibrium only for the locus g.98793763G>C, which was also characterized by greatest genetic diversity. The values of parameters for this locus, such as the effective number of alleles, gene heterozygosity, and polymorphic information content, were also highest (Table 1). 

It was determined that only the locus g.98793763G>C was associated, to a significant degree, with the antioxidant capacity of milk. Cows of the CC genotype produced milk of significantly higher antioxidant capacity as compared to cows of the GC genotype. It was also determined that the antioxidant capacity of milk varies significantly according to age of cows. The antioxidant capacity of milk from the oldest cows was significantly higher as compared to milk from younger animals. None of the analyzed loci of SNPs in the *LCN2* gene nor the age of cows had any impact on the daily milk yield, percentage of fat, protein, lactose, and SCC in milk (Table 2).

An analysis of the association between milk traits and the antioxidant capacity of milk showed an inverse relationship between the TEAC level and the daily milk yield. An increase in the daily milk yield was accompanied by reduced antioxidant capacity of milk and cows with the greatest milk yield (more than 35 kg/day) produced milk of significantly lower antioxidant capacity while cows with the lowest daily milk yield (less than 25 kg/day) produced milk of significantly higher antioxidant capacity. It was also determined that milk with somatic cell count of between 200,000 and 400,000/mL had a significantly higher antioxidant capacity as compared to milk with somatic cell count below 200,000/mL (Table 3). On the other hand, the percentage of fat, protein, and lactose had no significant impact on the antioxidant capacity of milk.

## 4. Discussion

Pathological conditions in the body are often due the accumulation of free radicals (mostly reactive oxygen species (ROS)) and the resulting oxidative stress. An increased pool of free radicals and the predominance of oxidation processes in the body may cause damage to cells and affect substances contained in them. It is one of the causes of body aging processes and chronic disorders (due to damage to proteins, lipids, or DNA) such as neoplasms, diabetes, cardiovascular and neurodegenerative diseases, etc. [19,20]. The effects of ROS in a body are balanced by antioxidants, substances which limit the level of oxidation of molecules and cause the transformation of free radicals into inactive derivatives [21]. Milk is one of the essential food products in the human diet and not only does it contain many indispensable nutrients (e.g., proteins, omega-3 fatty acids, vitamins, and minerals), but it is also a good source of antioxidants [22]. The antioxidant capacity of milk is determined primarily by the presence of bioactive components, in particular vitamins A, C, and E, β-carotene, superoxide dismutase, catalase and glutathione peroxidase [23]. Apart from environmental factors, the level of antioxidants can also vary according to genetic parameters. The objective of an analysis of polymorphisms in genes which might impact the level of antioxidants in cows’ milk is to identify genetic markers, on one hand are related to milk yield and quality, and on the other hand enhance the immunity of animals to inflammatory processes (including in particular mastitis) [24]. The lipocalin-2 gene could be such a marker. The LCN2 protein is of crucial relevance to innate immunity. It binds extracellularly to bacterial siderophores which are complexed with iron, thus producing bacteriostatic effects and preventing bacteria from absorbing iron [25]. Lipocalin-2 also acts as a specific preventive antioxidant as result of its anti-oxidative properties which prevent non-protein accumulation of iron ions. During inflammatory reactions, LCN2 binds to released iron ions which contain unpaired electrons, thus providing protection against free-radical reactions [26,27]. Analysis of polymorphisms of the bovine lipocalin-2 gene showed that this gene had a significant impact on milk production traits [10]. Our studies showed that an analysis of genotypes within the locus g.98793763G>C of the lipocalin-2 gene may provide valuable information to select cows according to greater antioxidant capacity of milk produced by them. Such an association was determined for cows of the CC genotype at the locus in question (Table 2). It should also be pointed out that a higher level of antioxidants in milk may be the result of a response to an ongoing or recently experienced mastitis. The diagnosis of subclinical and clinical mastitis is established based, first of all, on an increased somatic cell count (SCC) in cows’ milk. In accordance with Directive 92/46/EEC of April 1992, it is assumed that SCC at a level of 400,000/mL defines the limit between milk obtained from a healthy mammary gland and milk originating from an udder affected by infection. Inflammatory reaction increases the level of leukocytes and mast cells in the udder, which promotes the production and release of ROS in response to the destructive effects of bacteria [28,29]. The study demonstrated that milk of somatic cell count between 200,000 and 400,000/mL had the greatest antioxidant capacity. This value was significantly different from that obtained for milk with somatic cell count below 200,000/mL. The greater antioxidant capacity of milk as a result of higher somatic cell count is probably correlated with the greater leukocyte count in milk as leukocytes produce a greater quantity of endogenous enzymatic and non-enzymatic anti-oxidative compounds to protect the udder cells against harmful effects of oxidative stress [30]. 

Interestingly, our analysis showed that high-yield cows (more than 35 kg/day) produced milk of definitely lower antioxidant capacity as compared to cows whose daily milk yield was less than 25 kg (Table 3). It seems that the assessment of antioxidant capacity of milk may provide cattle farmers with a method for the determination of the upper limit of milk yield, which would also define the conditions for obtaining a product of health-promoting qualities.

This study showed that the antioxidant capacity of milk increases with the age of cows (Table 2). This is attributable to the fact that the quantity of free radicals increases as the aging of a body progresses, and thus the body is “forced” to produce antioxidants in greater quantities in order to mitigate the effects of free radicals [31].

## 5. Conclusions

To summarize, producing milk of high quality, which is determined, inter alia, by the level of antioxidant capacity, should be the key objective of modern cattle farming in line with the current needs of consumers. Milk rich in natural antioxidants can be an important component of diet oriented towards the prevention of e.g., chronic diseases and the delay of the onset of signs of aging. An analysis of polymorphisms of the lipocalin-2 gene in cattle can be helpful in selecting animals which would produce milk of greater antioxidant capacity. Cows of the CC genotype at the locus g.98793763G>C of the *LCN2* gene produced milk of significantly higher antioxidant capacity as compared to cows of the GC and GG genotypes. The antioxidant capacity of milk also varied according to age of cows, daily milk yield, and somatic cell count in milk. The results of this study were shown, that genotyping in *LCN2* locus may be of use in breeding programs. The selection of cows and bulls for reproductive purposes by taking into consideration the desired genotype in *LCN2* locus, could significantly improve the quality of milk and milk products in the future. It is proper to pay attention to the age of the cows and the antioxidant activity in milk. Is it possible that cows are culled from herds too early? The results of this study should encourage further research involving higher numbers and other breeds of cattle. We hope that our study will help to demonstrate the usefulness of the lipocalin-2 gene as a genetic marker for milk quality in the future.

## Figures and Tables

**Table 1:** The frequency of alleles and genotypes and genetic diversity parameters of genotyped SNPs within the bovine *LCN2* gene.

Loci	Genotype Frequencies			MAF	χ^2^ (HWE)	Genetic Diversity Parameters		
						He	Ne	PIC
g.98793626A>G	AA	AG	GG	G	0.00	0.285	1.399	0.218
	0.686	0.284	0.030	0.172				
g.98793763G>C	GG	GC	CC	C	15.87 *	0.487	1.949	0.446
	0.414	0.337	0.249	0.418				
g.98793889G>A	GG	GA	AA	A	0.15	0.269	1.368	0.233
	0.710	0.260	0.030	0.160				

MAF—minor allel frequency, HWE—Hardy–Weinberg equilibrium. * *p* < 0.001. He—gene heterozygosity; Ne—effective allele numbers; PIC—polymorphism information content.

**Table 2:** Effects of *LCN2* genotypes and cow age on the antioxidant capacity and milk traits such as: daily milk yield, milk fat, and protein percentage and somatic cell count (SCC).

Effect	Classes		N	TEAC (µM TE)		Daily Milk Yield (kg) *		Fat (%) *		Protein (%) *		Lactose (%) *		SCC * (×10^3^ cells/mL)	
				LSM	SE	LSM	SE	LSM	SE	LSM	SE	LSM	SE	LSM	SE
*LCN2* genotypes	g.98793626A>G	AA	116	3.53	0.25	26.23	0.73	3.75	0.07	3.52	0.03	4.60	0.03	512.88	71.80
		AG	48	2.97	0.38	26.64	1.12	3.83	0.11	3.60	0.05	4.71	0.04	303.28	110.71
		GG	5	2.67	1.17	25.94	3.44	3.80	0.33	3.40	0.16	4.66	0.13	307.62	338.81
	g.98793763G>C	GG	70	3.35	0.31	26.66	0.93	3.67	0.09	3.54	0.04	4.68	0.04	449.25	92.01
		GC	57	2.60 ^A^	0.35	26.14	1.04	3.79	0.10	3.59	0.05	4.67	0.04	443.62	103.87
		CC	42	4.30 ^A^	0.39	26.03	1.17	3.92	0.11	3.46	0.05	4.57	0.05	452.26	117.21
	g.98793889G>A	GG	120	3.48	0.24	26.26	0.71	3.77	0.07	3.53	0.03	4.61	0.03	513.22	69.92
		GC	44	3.06	0.41	26.60	1.19	3.79	0.11	3.58	0.06	4.72	0.05	271.92	117.21
		CC	5	2.67	1.17	25.94	3.44	3.80	0.33	3.40	0.16	4.66	0.13	306.93	338.15
age (year)		≤4	12	1.25 ^A^	0.73	23.77	2.18	4.06	0.21	3.48	0.11	4.78	0.09	326.33	216.21
		4.1–6.0	101	3.07 ^b^	0.25	26.75	0.75	3.77	0.07	3.56	0.04	4.64	0.03	446.54	74.52
		>6.0	56	4.07 ^Ab^	0.34	25.96	1.01	3.80	0.10	3.57	0.05	4.64	0.04	471.86	100.08

TEAC—the Trolox equivalent antioxidant capacity method, SCC—somatic cell count. * The average values for the lactation in which the antioxidant capacity of milk was measured. LSM—least-squares mean, SE—standard error. LSMs with the same superscript letters in the same column within the effect differ significantly at: ^A^ *p* < 0.01; ^b^ *p* < 0.05.

**Table 3:** Effects of milk traits on the antioxidant capacity of milk.

Effect	Classes	N	TEAC (µM TE)	
			LSM	SE
daily milk yield (kg) *	≤25.0	73	4.11 ^Ab^	0.29
	25.1–35.0	72	3.01 ^bc^	0.29
	>35.0	24	1.49 ^Ac^	0.50
fat (%) *	≤3.2	80	3.90	0.53
	3.21–4.0	73	3.82	0.38
	>4.0	16	3.27	0.37
protein (%) *	≤3.5	33	3.47	0.31
	3.5–4.0	73	3.20	0.34
	>4.0	63	4.33	0.70
lactose (%) *	≤4.40	30	3.47	0.54
	4.41–4.80	80	3.65	0.35
	>4.80	59	3.88	0.37
SCC (×10^3^ cells/mL) *	<200	82	2.82 ^b^	0.38
	200–400	36	4.00 ^b^	0.46
	>400	51	3.16	0.42

TEAC—the Trolox equivalent antioxidant Ccpacity method. * Measured on the day of milk sampling. LSM—least-squares mean, SE—standard error. LSMs with the same superscript letter differ significantly at: ^A^ *p* < 0.0001, ^b,c^ *p* < 0.05.
